# Repression of Noxa by Bmi1 contributes to deguelin‐induced apoptosis in non‐small cell lung cancer cells

**DOI:** 10.1111/jcmm.13908

**Published:** 2018-09-25

**Authors:** Wei Li, Xinfang Yu, Zhenkun Xia, Xinyou Yu, Li Xie, Xiaolong Ma, Huiling Zhou, Lijun Liu, Jian Wang, Yifeng Yang, Haidan Liu

**Affiliations:** ^1^ Department of Cardiovascular Surgery The Second Xiangya Hospital of Central South University Changsha Hunan China; ^2^ Department of Radiology The Third Xiangya Hospital of Central South University Changsha Hunan China; ^3^ Department of Cancer Biology Lerner Research Institute Cleveland Clinic Cleveland Ohio; ^4^ Department of Thoracic Surgery The Second Xiangya Hospital of Central South University Changsha Hunan China; ^5^ Shangdong Lvdu Bio‐Industry Co., Ltd. Binzhou Shangdong China; ^6^ Clinical Center for Gene Diagnosis and Therapy The Second Xiangya Hospital of Central South University Changsha Hunan China

**Keywords:** apoptosis, Bmi1, deguelin, Noxa, NSCLC

## Abstract

Deguelin, a natural rotenoid isolated from several plants, has been reported to exert anti‐tumour effects in various cancers. However, the molecular mechanism of this regulation remains to be fully elucidated. Here, we found that deguelin inhibited the growth of non‐small cell lung cancer (NSCLC) cells both in vitro and in vivo by downregulation of Bmi1 expression. Our data showed that Bmi1 is highly expressed in human NSCLC tissues and cell lines. Knockdown of Bmi1 significantly suppressed NSCLC cell proliferation and colony formation. Deguelin treatment attenuated the binding activity of Bmi1 to the *Noxa* promoter, thus resulting in *Noxa* transcription and apoptosis activation. Knockdown of Bmi1 promoted Noxa expression and enhanced deguelin‐induced apoptosis, whereas overexpression of Bmi1 down‐regulated Noxa protein level and deguelin‐induced apoptosis. Overall, our study demonstrated a novel apoptotic mechanism for deguelin to exert its anti‐tumour activity in NSCLC cells.

## 1 INTRODUCTION

Lung cancer is the leading cause of cancer‐related death worldwide. There are two main categories of lung cancers: small cell lung cancer and non‐small cell lung cancer (NSCLC). NSCLC accounts for 80% of all lung tumours.^1^ Although chemotherapy can relieve symptoms and improve survival rate, the 5‐year survival rate of patients with NSCLC is low.^2^ Finding new targets and novel anti‐cancer agents with fewer side effects will provide more effective strategies for NSCLC treatment.

B cell‐specific Moloney murine leukaemia virus integration site 1 (Bmi1) is thought to be a critical component of the polycomb repressive complex 1 (PRC1), including a number of proteins (Bmi1, Ring1, HPH1, HPC1, and HPC2). Bmi1 is an epigenetic regulator for the stable maintenance of gene repression^3^ that involved in the regulation of development, stem cell self‐renewal, cell cycle, senescence, and tumourigenesis.^4^, ^5^, ^6^, ^7^ Studies have found that Bmi1 regulates cell senescence and proliferation via transcriptional silencing of tumour suppressor genes, such as *p16* ^*INK4a*^, *p19* ^*ARF*^, and *p21* ^*Cip1*^, and results in tumourigenesis.^6^, ^7^, ^8^ In addition, Bmi1 is known to inhibit other tumour suppressor genes, including *PTEN*,^9^ *BCL2L11*,^10^, ^11^ and *WWOX*.^12^ A recent study has revealed that Bmi1 regulates memory CD4 T cell survival and function through the direct repression of *Noxa* gene.^13^ Teshima et al^11^ report that Bmi1 directly regulates pro‐apoptotic genes such as *BCL2L11/Bim* and *PMAIP1/Noxa*, leading to enhance anti‐apoptotic potential of mantle cell lymphoma.

It has been reported that Bmi1 functions as an oncogene. Aberrant overexpression of Bmi1 has been reported in multiple tumour types, including breast cancer,^14^ colon carcinoma,^15^ melanoma,^16^ and hepatocellular carcinoma.^17^ Although upregulation of Bmi1 in human NSCLC has been reported,^18^ the role of Bmi1 in the pathogenesis of NSCLC and its exact target genes have not been extensively studied. Accumulation of evidence has demonstrated that Bmi1 plays a critical role in cancer cell invasion, metastasis, and chemoresistance.^19^ Overexpression of Bmi1 correlates with cancer development, progression, and therapy failure.^17^, ^20^, ^21^ Moreover, experimental decrease of Bmi1 protein levels results in cancer cells apoptosis and/or senescence, increasing susceptibility to cytotoxic agents and radiation therapy.^22^, ^23^ These data suggest that reducing Bmi1 protein level may have a beneficial effect in multiple types of cancer including NSCLC.

*Noxa*, a pro‐apoptotic BH3‐only member of the Bcl‐2 family of proteins,^24^ albeit showing weak pro‐apoptotic potential on its own, appears to be crucial in fine‐tuning cell death decisions by targeting the pro‐survival molecule Mcl‐1 for proteasomal degradation.^25^ *Noxa* expression is traditionally known to be modulated by p53‐dependent mechanisms.^24^, ^26^ Many p53‐independent mechanisms of Noxa upregulation have been identified. For instance, the transcription factors c‐Myc,^27^ HIF‐1α,^28^ CREB^29^ and E2F1^30^ have been described to mediate p53‐independent transcription of *Noxa*. Furthermore, recent studies have shown that Bmi1 suppresses *Noxa* expression in memory CD4 T cells and mantle cell lymphoma.^11^, ^13^ However, the mechanisms underlying Noxa induction and the functional significance of Noxa in NSCLC have not been studied.

Deguelin is a natural rotenoid extracted from several plants, including *Derris trifoliata* Lour (Leguminosae), *Mundulea sericea* (Leguminosae). It has shown great potential as a cancer chemopreventive and therapeutic agent for various types of cancer, including lung and breast cancers.^31^ Deguelin has been reported to induce cell apoptosis through inhibiting many signalling pathways, such as PI3K/Akt/HK2,^32^, ^33^ IKK/IκBα/NF‐κB,^34^ and AMPK/mTOR/survivin.^35^ Additionally, the anti‐cancer effect has been associated with many other mechanisms, including inhibition of tumour cell propagation and malignant transformation through p27/cyclinE/pRb/E2F1 or Aurora B for cell cycle control,^36^, ^37^, ^38^, ^39^ HIF‐1α/VEGF and HGF/c‐Met for anti‐angiogenic,^40^, ^41^ and GSK‐3β/β‐catenin for anti‐metastasis.^42^ These findings suggest that deguelin functions as an anti‐tumourigenic agent targeting apoptosis, cell cycle arrest and anti‐angiogenesis for cancer therapeutic intervention. Thus, the mechanism by which deguelin induces apoptosis in human cancers including NSCLC need to be fully revealed.

In this study, we investigated the underlying mechanism of deguelin‐induced apoptosis in NSCLC cell lines. Our results demonstrate that deguelin inhibits the growth of NCSLC cells both in vitro and in vivo by down‐regulating Bmi1 expression and thus relieving Bmi1‐mediated Noxa repression, finally leading to NSCLC cells apoptosis. Bmi1‐mediated Noxa repression is achieved through the direct binding of Bmi1 to the *Noxa* promoter in NSCLC cells. Deguelin attenuates the binding of Bmi1 to the *Noxa* promoter and removes Bmi1‐caused repression, resulting in Noxa induction. This study provides a novel mechanism for deguelin exerting inhibitory effects on NSCLC cell, which is related to the suppression of Bmi1.

## 2 MATERIALS AND METHODS

### 2.1 Reagents and plasmid constructs

Deguelin (>97% purity) and other chemical reagents, including Tris, NaCl, SDS, and DMSO, for molecular biology and buffer preparation, were purchased from Sigma‐Aldrich (St. Louis, MO, USA). z‐VAD‐fmk (cat#S7023), Necrostatin‐1 (cat#S8037), and GSK'872 (cat#S8465) were purchased from Selleckchem (Houston, TX, USA). Lentivirus plasmids containing *pLKO.1‐shBmi1* (#1, TRCN0000020154; #2, TRCN0000020155; #3, TRCN0000020156; #4, TRCN0000020157; #5, TRCN0000020158) were purchased from Thermo Scientific (Rockford, IL, USA), *pLKO.1‐shNoxa* (V3SH11240‐224893462) was purchased from GE Dharmacon (Lafayette, CO, USA). The Bmi1 expression construct *pT3‐EF1a‐Bmi1* (#31783), the luciferase reporter *pGL3‐Noxa‐N1* (#26112), *pLKO.1‐shGFP* (#30323), the lentiviral packaging plasmid *psPAX2* (#12260), and the envelope plasmid *pMD2.G* (#12259) were available on Addgene (Cambridge, MA, USA). The *pGL3‐Basic* and the *Renilla* luciferase reporter construct *pRL‐SV40* (Promega, Madison, WI, USA) was used as previously described.^43^

### 2.2 Cell lines and cell culture

Cells from American Type Culture Collection (ATCC) were cultured at 37°C in a humidified incubator with 5% of CO_2_ according to the ATCC protocols. Cells were cytogenetically tested and authenticated before being frozen. Each vial of frozen cells was thawed and maintained for 2 months (10 passages). Of note, 293T cells were cultured with Dulbecco's Modified Eagle Medium containing 10% of FBS and 1% of antibiotics. Human NSCLC cells, including NCI‐H1299, NCI‐H460, NCI‐H520, NCI‐H23, and NCI‐H125, were grown in RPMI‐1640 medium supplemented with 10% of FBS and 1% of antibiotics. A549 human NSCLC cells were cultured with F‐12K medium containing 10% of FBS and 1% of antibiotics. MRC5 human normal lung fibroblasts were cultured with Eagle Minimum Essential Medium supplemented with 10% of FBS and 1% of antibiotics. The cells were cultured for 36‐48 hours and proteins extracted for analysis.

### 2.3 Clinical tissue sample collections

Fresh tumour tissues and the corresponding normal adjacent tissues of the same patient with pathologically and clinically confirmed NSCLC by the Department of Clinicopathologic were collected from 22 patients with written informed consent by the Department of Thoracic Surgery, The Second Xiangya Hospital of Central South University, Changsha, Hunan, China. Several small pieces of fresh tumour tissue samples were dissected from the main tumour part of each surgically removed specimen. A portion of tumour and normal adjacent tissues were frozen immediately in liquid nitrogen and then stored at −80°C for protein extraction and analysis of protein expressions by Western blotting. A portion of tumour and normal adjacent tissues were fixed in formalin solution and sent for histological examination. Prior patient consent and approval from the Hospital's Research Ethics Committee were obtained for the use of these clinical materials for research purposes. All the patients received no treatment before surgery.

### 2.4 Lentiviral infection and transient transfection

The generation of gene stable knockdown cell lines was performed as described previously.^44^ Briefly, to generate Bmi1 knockdown cells, *pLKO.1‐shGFP,* and *pLKO.1‐shBmi1* lentivirus plasmids were co‐transfected into 293T cells with *psPAX2* and *pMD2.G*. Viral supernatant fractions were collected at 48 hours after transfection and filtered through a 0.45 μm filter followed by infection into NCI‐H23, NCI‐H1299, or NCI‐H460 cells together with 6 μg/mL polybrene. At 16 hours after infection, the medium was replaced with fresh medium containing 2 μg/mL puromycin and cells were incubated for another 3 days. For transient transfection, NSCLC cells growing on 24‐well plates were transfected with the *pGL3‐Noxa‐N1* plasmid or the *pGL3‐Basic* vector overnight then treated with various concentration of deguelin for 48 hours; or NSCLC cells were co‐transfected the *pGL3‐Noxa‐N1* plasmid or the *pGL3‐Basic* vector along with *pT3‐EF1a‐Bmi1* or empty vector for 48 hours using Lipofectamin 2000 (cat#11668‐019; Invitrogen, Carlsbad, CA, USA) following the manufacturer's instructions. Each transfection was contained the *Renilla* luciferase reporter construct *pRL‐SV40*. Firefly luciferase and *Renilla* luciferase activity was determined using the Dual‐Luciferase reporter assay system (#E1910; Promega, Madison, WI, USA) with a GloMax 20/20 luminometer (#E5311; Promega, Madison, WI, USA). Firefly luciferase readings were normalized to *Renilla* luciferase to correct for transfection efficiency. The data are represented as the fold induction compared to the *pGL3‐Basic* vector. All experiments were performed in triplicate with at least two independent experiments.

### 2.5 Protein preparation and Western blotting

Protein preparation and Western blotting were performed according to the method previously described.^44^ The mitochondrial fraction was prepared using the Mitochondria Isolation Kit (cat#89874; Thermo Scientific, Rockford, IL, USA) according to the instructions provided. Protein concentration was determined using the BCA Assay Reagent (cat#23228; Pierce, Rockford, IL, USA). Western blotting was performed as previously described.^44^ Primary antibodies were used for immunoblotting: Bmi1 (#6964), Mcl‐1 (#5453), Bcl‐2 (#2870), Bcl‐xL (#2764), cleaved caspase‐3 (#9664), cleaved caspase‐9 (#9505), cleaved PARP (#5625), and Tri‐Methyl‐Histone H3 (Lys27) (#9733) from Cell Signaling Technology (Danvers, MA, USA); Tri‐Methyl‐Histone H3 (Lys9) (#07‐) from Millipore (Burlington, MA, USA); Noxa (#OP180) from Merck Millipore (Darmstadt, Germany); p53 (sc‐126) from Santa Cruz Biotechnology (Dallas, TX, USA); β‐actin (A5316) from Sigma‐Aldrich (St. Louis, MO, USA). Secondary antibodies were anti‐rabbit IgG HRP (#7074) and anti‐mouse IgG HRP (#7076) and purchased from Cell Signaling Technology (Danvers, MA, USA). Antibody conjugates were visualized by chemiluminescence (ECL; cat#34076; Thermo, Rockford, IL, USA).

### 2.6 Cell proliferation assays

Cells were seeded at a density of 2 × 10^3^ cells per well in 96‐well plates in 100 μL of RPMI 1640 medium containing 10% of FBS without or with different concentrations of deguelin and incubated in a 37°C, 5% of CO_2_ incubator. After culturing for 24, 48, 72, or 96 hours, 10 μL of the WST‐1 reagent (#11644807001; Roche, Mannheim, Germany) were added to each well and cells were incubated for 2 hours at 37°C. The absorbance of the cellular reduction of WST‐1 to formazan was measured at 450 nm as previously described.^44^ Three independent experiments were performed in triplicate.

### 2.7 Anchorage‐independent cell growth assay

Cells (8 × 10^3^ per well) were suspended in basal medium Eagle (1 mL with 10% of FBS and 0.3% of agar) in the absence or presence of various concentrations of deguelin and plated over a layer of solidified basal medium Eagle (1 mL with 10% of FBS and 0.5% of agar) without or with various concentrations of deguelin. The cultures were maintained at 37°C in a 5% of CO_2_ incubator for 2 or 3 weeks and colonies were counted under a microscope as previously described.^44^

### 2.8 Immunohistochemistry

Tumour tissues obtained from euthanized xenografted mice were embedded in paraffin and subjected to immunohistochemical staining with specific antibodies against Bmi1 (1:50, #6964; Cell Signaling Technology, Danvers, MA, USA), Noxa (1:100, ab36833; Abcam, Cambridge, MA, USA), cleaved caspase‐3 (1:50, #9664; Cell Signaling Technology, Danvers, MA, USA), or Ki67 (1:200, ab16667; Abcam, Cambridge, MA, USA) according to the DAKO system protocol. Hematoxylin was used for counterstaining. Slides were viewed and photographed under a light microscope. The integrated optical density was quantified using Image‐Pro Plus Software (version 6.2) program (Media Cybernetics, Rockville, MD, USA) as previously described.^45^

### 2.9 Chromatin‐immunoprecipitation assay

Chromatin‐immunoprecipitation (ChIP) assays were performed as previously described.^44^ Briefly, the deguelin‐treated NSCLC cells were cross‐linked with 1% of formaldehyde, neutralized with 125 mM glycine, harvested, and disrupted by sonication to fragments with an average size of ~500 bp. The chromatin of cells was pre‐cleared with 30 μL protein G agarose/salmon sperm DNA (#16‐201; Upstate, Temecula, CA, USA) and incubated with 2 μg of Bmi1 (#6964; Cell Signaling Technology, Danvers, MA, USA), Tri‐Methyl‐Histone H3 (Lys27) (#9733; Cell Signaling Technology, Danvers, MA, USA), or normal rabbit IgG (#NI01; Calbiochem, Darmstadt, Germany) antibody at 4°C overnight. The immunocomplexes were pulled down with 30 μL dynabeads Protein G (#100.03D; Invitrogen, Carlsbad, CA, USA). The beads were collected on a magnetic device and washed with ChIP wash buffer and TE buffer (10 mM Tris‐HCl, pH 8.0, 1 mM EDTA). Cross‐links for both ChIP and input DNA were reversed at 65°C for 5 h and DNA was purified with E.Z.N.A Cycle‐pure Kit (Omega BIO‐TEK, Norcross, GA, USA). Equal amount of each ChIP‐DNA was used as a template for polymerase chain reactions (PCR). PCR products were analysed by electrophoresis on a 3% of agarose gel and visualized by ethidium bromide staining. The primer pairs (Table S1) were used to amplify the *Noxa* promoter regions present in the immunoprecipitated DNA.

### 2.10 In vivo tumour growth assay

All animal procedures were approved by the Institutional Animal Care and Use Committee of the Second Xiangya Hospital, Central South University, China. Xenograft tumours were established by s.c. injection of NCI‐H1299 (3 × 10^6^) or A549 cells (3 × 10^6^) into the flank of 6‐week‐old athymic nude mice (n = 12). When tumours reached an average volume of 100 mm^3^, treatment with deguelin (3 mg/kg), or vehicle was initiated and repeated daily by i.p. injection. Mice were weighed and tumours measured by caliper every 2 days. Tumour volume was calculated from measurements of 2 diameters of the individual tumour according to the following formula: tumour volume (mm^3^) = (length × width × width/2).

### 2.11 Blood analysis

Mouse blood was collected in EDTA‐coated tubes via cardiac puncture of anaesthetized mice for hematology studies. The white blood cells (WBC), hemoglobin (Hb), red blood cells (RBC), aspartate aminotransferase (AST), alanine aminotransferase (ALT), and blood urea nitrogen (BUN) were analysed in the Clinical Laboratory at the Second Xiangya Hospital of Central South University (Changsha, Hunan, China).

### 2.12 Statistical analysis

Statistical analysis was performed with SPSS 16.0 (SPSS Inc, Chicago, IL, USA). Results expressed as mean ± SD were analysed using the Student's *t* test. Differences were considered significant when *P* < 0.05.

## 3 RESULTS

### 3.1 Bmi1 is overexpressed in human NSCLC

**Figure 1:**
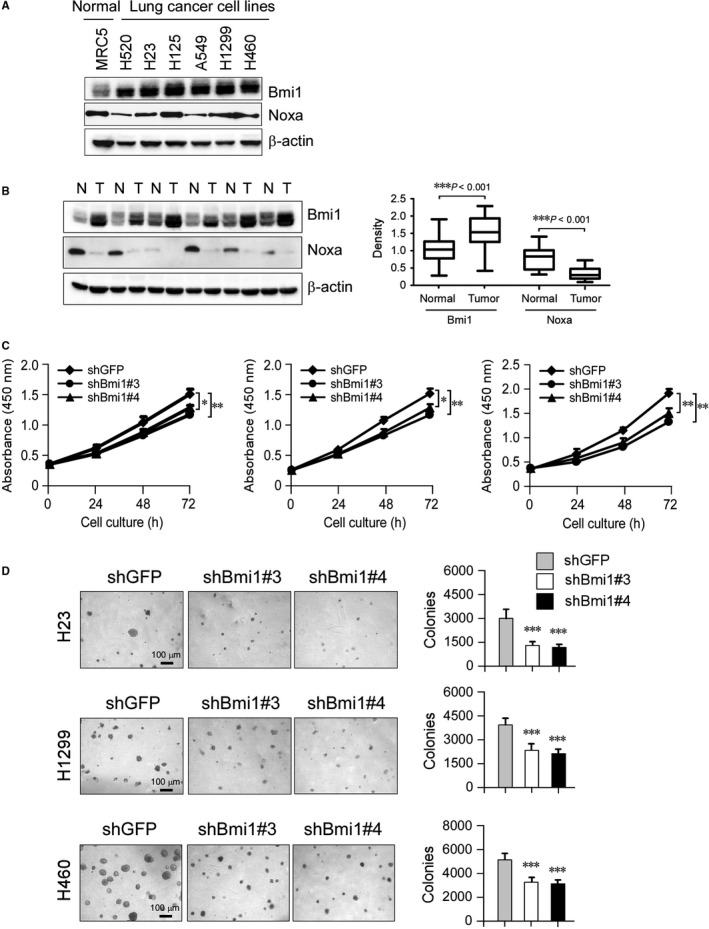
Expressions of Bmi1 and Noxa in human non‐small cell lung cancer. A, Western blot analysis was performed to examine Bmi1 and Noxa expressions in several NSCLC cell lines and normal MRC5 lung cells. β‐actin was used as a loading control. B, Bmi1 and Noxa protein levels in six representative NSCLC cases was assessed by Western blot analysis. β‐actin was used as a loading control. N, normal adjacent tissue; T, tumour (*left panel*). Western blotting determined Bmi1 and Noxa protein levels in the malignant and the corresponding normal adjacent tissues of 22 NSCLC patients (*right panel*). The intensity was evaluated using Image J (NIH) computer software. ***P < 0.001, significant difference between groups as indicated. C, knockdown of Bmi1 attenuated NCI‐H23, NCI‐H1299, and NCI‐H460 anchorage‐dependent cell growth. WST‐1 assays were performed as described in Materials and Methods. Data represent mean ± SD from three independent experiments. *P < 0.05, **P < 0.01, significant difference compared with the shGFP control cells. D, Knockdown of Bmi1 attenuated NCI‐H23, NCI‐H1299, and NCI‐H460 anchorage‐independent cell growth. Soft agar assays were performed as described in Materials and Methods. Data represent mean ± SD from two independent experiments. ***P < 0.001, significant difference compared with the shGFP control cells

To investigate whether the expression of Bmi1 and Noxa were linked to human NSCLC, Bmi1 and Noxa protein levels were examined by Western blotting in cultures of human fetal lung fibroblast cells and six human NSCLC cell lines. When compared to fetal lung fibroblast cells, Bmi1 was expressed at higher levels in all human NSCLC cell lines tested, however, Noxa was substantially down‐regulated in human NSCLC cell lines (Figure 1A). We next sought to examine the expression of Bmi1 in human NSCLC and matched normal adjacent tissue specimens. In matched normal adjacent samples, Bmi1 was expressed at a relatively low level. On the contrary, Bmi1 was significantly higher in the group of tumour samples (Figure 1B). Importantly, Noxa was highly expressed in normal adjacent tissues but dramatically decreased in tumour tissues (Figure 1B). Furthermore, knockdown of Bmi1 (Figure S1) inhibited both anchorage‐dependent (Figure 1C) and ‐independent cell growth (Figure 1D) in NCI‐H23, NCI‐H1299, and NCI‐H460 cells, suggesting that blocking Bmi1 expression reduced the tumourigenic properties of NSCLC cells in vitro. These results indicate that the PRC1 component Bmi1 and Noxa play critical roles in NSCLC.

### 3.2 Deguelin inhibits anchorage‐dependent and ‐independent growth of human NSCLC cells

**Figure 2:**
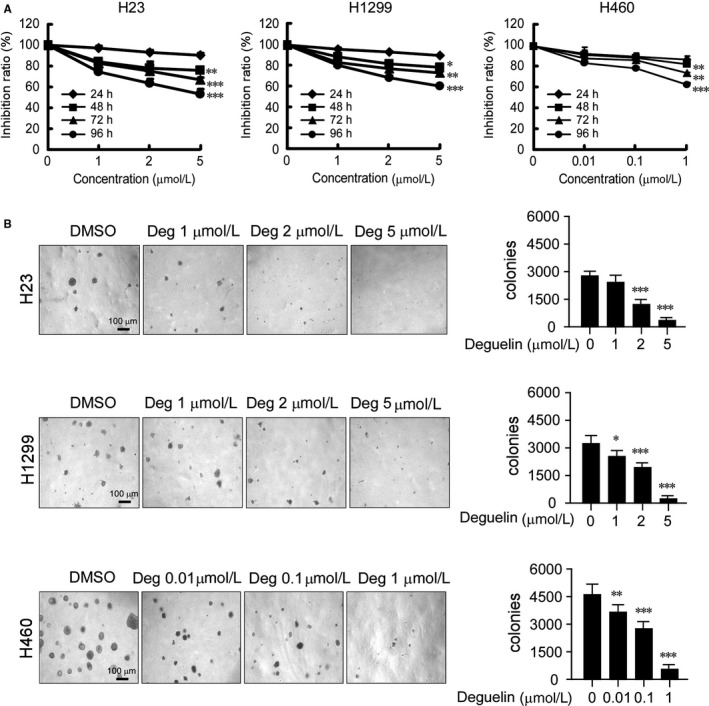
Deguelin inhibits anchorage‐dependent and ‐independent growth of NSCLC cells. A, Deguelin suppresses anchorage‐dependent growth of NSCLC cells. NCI‐H23, NCI‐H1299, and NCI‐H460 NSCLC cells were treated with DMSO or the indicated concentrations of deguelin in medium containing 10% of FBS and growth was measured at the indicated times using the WST‐1 assay. Data represent mean ± SD from three independent experiments. *P < 0.05, **P < 0.01, ***P < 0.001, significant difference compared with the DMSO control cells. B, Deguelin suppresses anchorage‐independent growth of NSCLC cells. Soft agar assay was performed as described in Materials and Methods. The cultures were incubated for 14 days and then colonies were counted. Data represent mean ± SD from two independent experiments. *P < 0.05, **P < 0.01, ***P < 0.001, significant difference compared with the DMSO control cells

Deguelin has shown potential chemopreventive and chemotherapeutical activities against various human cancers. In the present study, we first examined the effect of deguelin on the anchorage‐dependent growth of NSCLC cell lines, including NCI‐H23, NCI‐H1299, and NCI‐H460 cells. We found that deguelin significantly inhibited the growth of all tested cell lines in a dose‐dependent manner (Figure 2A). Notably, we observed that H460 cells were far more sensitive to deguelin and all died at 2 μM concentration (data not shown). Therefore, the maximum concentration of deguelin for H460 exposure in the present study was 1 μM. Meanwhile, the inhibitory effect of deguelin on anchorage‐independent growth was examined in a soft agar medium. The result showed that deguelin significantly suppressed the colony formation of NSCLC cells in soft agar (Figure 2B). The number of colonies formed was reduced in a dose‐dependent manner. In addition, the majority of the colonies in the deguelin‐treated group were smaller than those in the vehicle‐treated control (Figure 2B). The results indicate that deguelin suppresses both anchorage‐dependent and ‐independent growth of NSCLC cells.

### 3.3 Deguelin inhibits the growth of NSCLC cells in a xenograft mouse model

**Figure 3:**
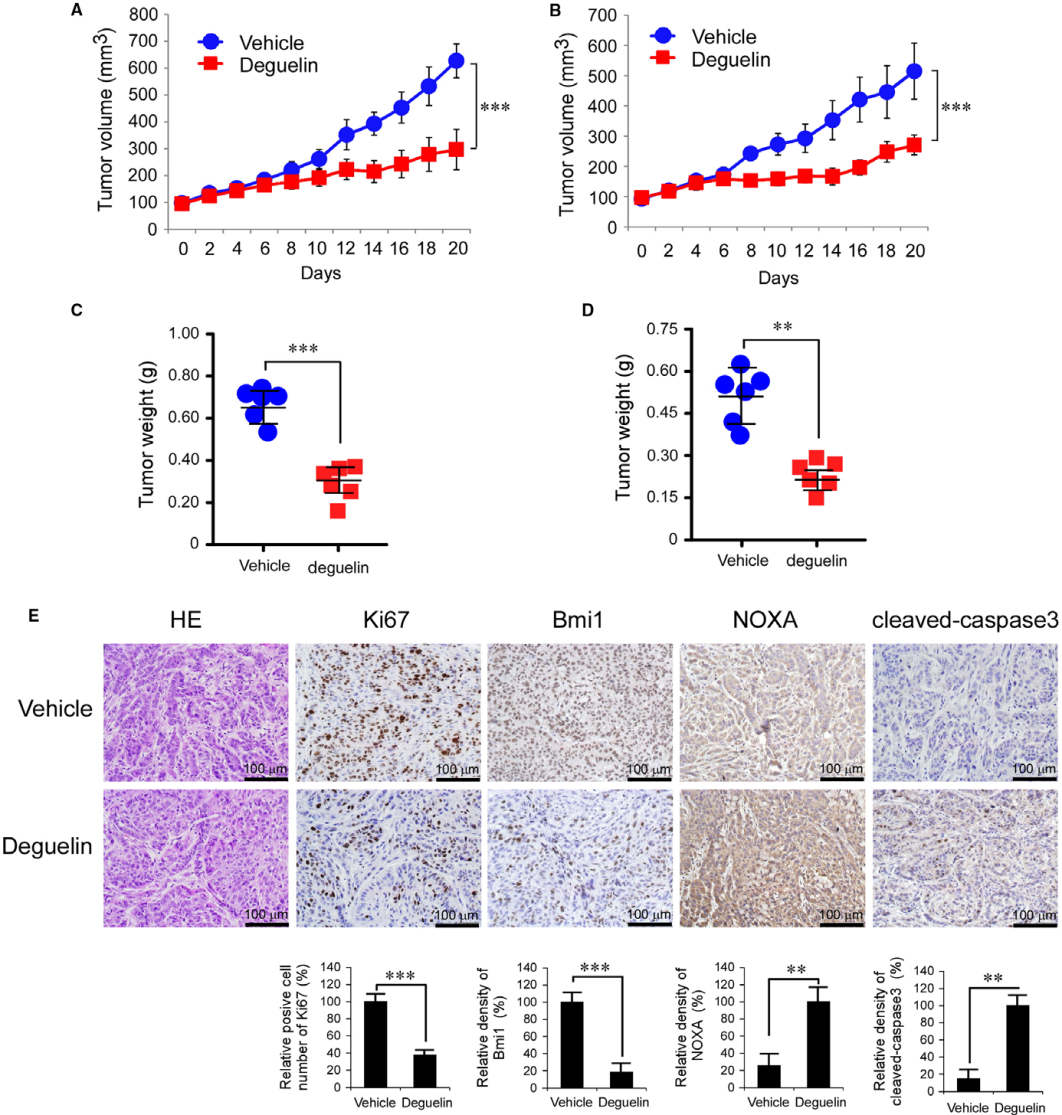
Deguelin suppresses tumour growth in vivo. A and B, Deguelin significantly inhibited tumour growth in H1299 (A) and A549 (B) xenograft mouse models. C and D, The tumour weight from vehicle‐ and deguelin‐treated group was measured in H1299 (C) and A549 (D) xenograft mouse models. E, Immunohistochemical staining examination of Ki67, Bmi1, Noxa, and cleaved caspase‐3 in H1299 tumour sections from the mice of the vehicle‐ or deguelin‐treated group. All panels are of the same magnification (**P < 0.01, ***P < 0.001, vs vehicle‐treated group)

To determine the inhibitory effect of deguelin in vivo, we explored NCI‐H1299 and A549 athymic nude xenograft mouse models. Data showed that deguelin significantly inhibited tumour growth in both H1299 (Figure 3A and C, Figure S2A) and A549 (Figure 3B and D, Figure S2B) xenograft models. In vehicle‐treated group, the average tumour volume of H1299 (Figure 3A) and A549 (Figure 3B) reached 628 ± 63 mm^3^ and 515 ± 93 mm^3^, respectively. In deguelin treated group, the average tumour volume was only 297 ± 75 mm^3^ and 271 ± 33 mm^3^ (*P* < 0.001), respectively. Additionally, deguelin dramatically decreased tumour weight in these xenograft models (Figure 3C and D). Immunohistochemical analysis was performed to evaluate the expression levels of Ki67, Bmi1, Noxa, and cleaved caspase‐3 in the H1299 xenograft tumour. Results showed that Ki67 and Bmi1 were significantly inhibited in deguelin‐treated group. The possibility that the decrease in Ki67 is due to the relief of repression on the transcriptional targets of Bmi1, such as *p16* ^*INK4a*^, *p19* ^*ARF*^, and *p21* ^*Cip1*^,^6^, ^7^, ^8^ by deguelin‐attenuated Bmi1, cannot be excluded at this time. However, deguelin induced the upregulation of Noxa and cleaved caspase‐3, which indicated that treatment with deguelin may cause cell apoptosis in tumour (Figure 3E). Meanwhile, no obvious toxicity was observed as evaluating the change of body weight of tumour‐bearing mice between the vehicle‐ and the deguelin‐treated groups (Figure S2C and D). In order to further evaluate the in vivo toxicity of deguelin, the blood analysis was conducted. Data showed that deguelin treatment has no obvious effect on WBC and RBC count (Figure S2E). Moreover, the expression of Hb, ALT, AST, and BUN were consistent in vehicle‐ and deguelin‐treated groups (Figure S2E), which indicated that deguelin had no significant toxicity to vital organ functions and no hematologic toxicities. H&E staining also showed there is no detectable toxicity in normal tissue (heart, liver, spleen, lung, and kidney) in deguelin‐treated animals (Figure S2F). The results imply that deguelin is a well‐tolerated compound at the dose of 3 mg/kg, and the inhibitory effect of deguelin on the xenograft tumour growth may partly depend on the suppression of Bmi1 and the induction of Noxa expression.

### 3.4 Suppression of Bmi1 level by deguelin accompanies increased Noxa expression and apoptosis in NSCLC cells

**Figure 4:**
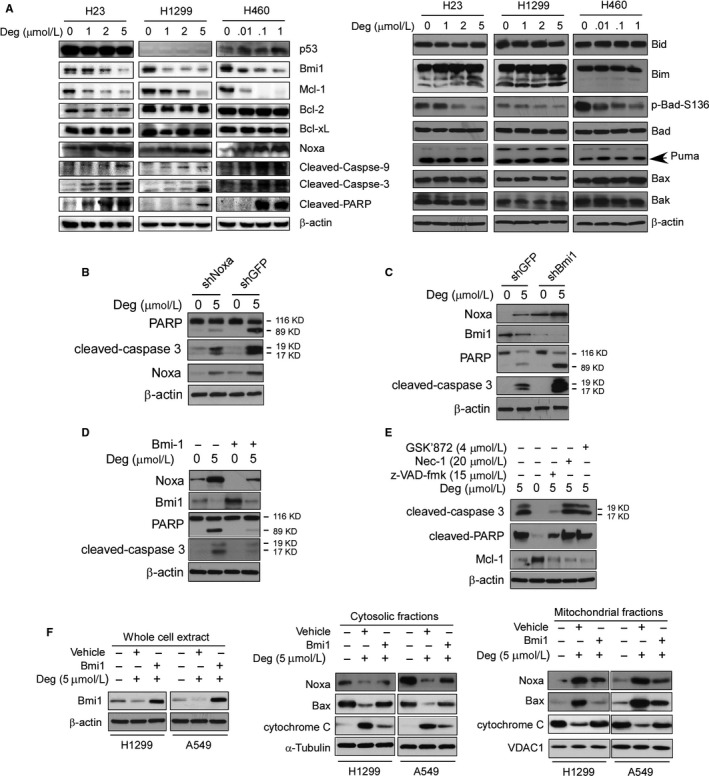
Effect of deguelin on the expression of apoptotic‐related proteins in NSCLC cells. A, NCI‐H23, NCI‐H1299, and NCI‐H460 NSCLC cells were treated with DMSO or the indicated concentrations of deguelin in medium containing 10% of FBS for 48 hours. After treatment, attached and floating cells were harvested. Expression of the indicated proteins was analysed by Western blotting with specific antibodies. β‐actin was used as a loading control. B, H1299‐shGFP, and H1299‐shNoxa stable cells were treated with deguelin as indicated, whole cell extract was analysed by Western blotting with specific antibodies. C, H1299‐shGFP, and H1299‐shBmi1 stable cells were treated with deguelin as indicated, whole cell extract was analysed by Western blotting with specific antibodies. D and E, H1299 cells were treated with a panel of inhibitors as indicated, Western blotting was performed to detect apoptosis. F, H1299, and A549 cells were transfected with the Bmi1 plasmid, whole cell extract, cytosolic fractions, and mitochondrial fractions were subjected to Western blotting analysis with specific primary antibodies

Deguelin has been found to cause apoptosis in several cancer cell lines.^46^ Our data showed that deguelin‐suppressed Bmi1 was accompanied by the increases of Noxa and cleaved caspase‐3 staining in the H1299 xenograft tumour (Figure 3E), which suggested that the induction of apoptosis was involved in deguelin‐mediated NSCLC suppression. We thus analysed other apoptotic markers,^47^ such as cleaved caspase‐9 and cleaved poly (ADP‐ribose) polymerase (PARP), to check apoptosis upon deguelin treatment in NSCLC cells by immunoblotting in a panel of human NSCLC cell lines. The results showed that deguelin induced caspase‐9, caspase‐3, and PARP cleavage, indicating NCI‐H23, NCI‐H1299, and NCI‐H460 cells showed apoptosis upon deguelin treatment (Figure 4A, *left*). In addition, these results showed that Bcl‐2 and Bcl‐xL levels were unchanged at all‐time points tested, whereas Bmi1 and Mcl‐1 levels were decreased and Noxa level was increased after deguelin exposure in these NSCLC cell lines (Figure 4A, *left*). We further analysed the protein level of other Bcl‐2 family members, including Bid, Bim, Bad, Puma, Bax, and Bak. Results showed that deguelin treatment had no obvious effect on the total protein levels of these Bcl‐2 family members (Figure 4A, *right*). Since Bmi1 reportedly mediates Noxa repression,^11^, ^13^ these results suggest that deguelin might affect the Bmil/Noxa axis, through which contributes to deguelin‐induced apoptosis in these NSCLC cells. In order to verify this hypothesis, we constructed stable Noxa or Bmi1 knockdown cell lines. As shown in Figure 4B, knocking down of Noxa compromised deguelin‐induced apoptosis in H1299 cells. Additionally, decrease of Bmi1 promoted Noxa expression and enhanced deguelin‐induced apoptosis (Figure 4C), whereas overexpression of Bmi1 down‐regulated Noxa protein levels and impaired deguelin‐induced apoptosis significantly (Figure 4D). Pre‐treated H1299 cells with multiple apoptosis or necroptosis inhibitors, z‐VAD‐fmk, Necrostatin‐1 (Nec‐1), and GSK'872, we found that only z‐VAD‐fmk dramatically decreased deguelin‐induced cell death (Figure 4E). These results suggest that deguelin‐induced cell death is dependent on the activation of apoptosis signaling pathway, and the Bmi1/Noxa axis plays a crucial role in this process. In order to further confirm the inhibitory effect of Bmi1 on deguelin‐induced apoptosis, we overexpressed Bmi1 in H1299 and A549 cells (Figure 4F, *left*), mitochondrial fraction was isolated and subjected to Western blot analysis. These results showed that overexpression of Bmi1 suppresses deguelin‐induced Bax and Noxa mitochondrial localization and cytochrome c release from mitochondria (Figure 4F, *middle* and *right*). Current data suggest that downregulation of Bmi1 and overexpression of Noxa is involved in deguelin‐induced apoptosis in NSCLC cells.

### 3.5 Deguelin induces cell apoptosis through Bmi1‐mediated Noxa upregulation in NSCLC cells

**Figure 5:**
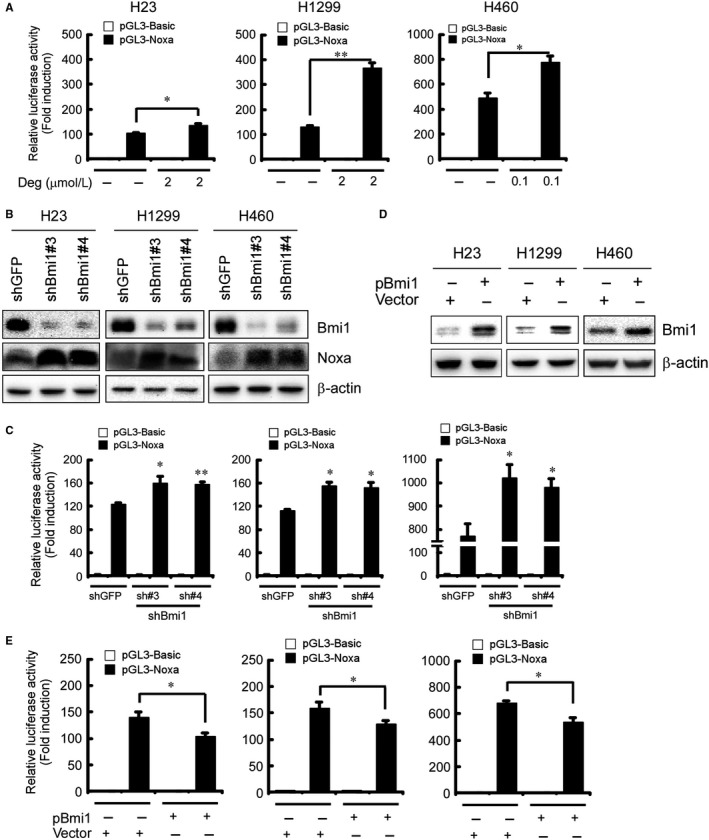
Involvement of Bmi1 in deguelin‐induced Noxa expression in NSCLC cells. A, Deguelin increases the *Noxa* promoter activity in NSCLC cells. Dual luciferase reporter assays of plasmid DNA encoding a fragment of human *Noxa* promoter in NSCLC cells were performed as described in Materials and Methods. NCI‐H23, NCI‐H1299, and NCI‐H460 cells were transfected with the *Noxa* promoter reporter plasmid (*pGL3‐Noxa‐N1*) or *pGL3‐Basi*c vector and then exposed to deguelin for 48 hours. Firefly luciferase readings were normalized to *Renilla* luciferase to correct for transfection efficiency. The *Noxa* promoter‐driven luciferase activities were expressed as fold induction over the activity of *pGL3‐Basic* vector. Data represent mean ± SD from two independent experiments performed in triplicate. *P < 0.05, **P < 0.01, significant difference compared with the DMSO control cells. B, Knockdown of Bmi1 up‐regulates Noxa protein level in NSCLC cells. Stable knockdown of PRC1 component Bmi1 in NCI‐H23, NCI‐H1299, and NCI‐H460 cells and the level of Bmi1 and Noxa were examined by Western blot analysis with specific antibodies. β‐actin was used as a loading control. C, Knockdown of Bmi1 increases the *Noxa* promoter activity in NSCLC cells. Stable Bmi1‐knockdown NCI‐H23 (*left panel*), NCI‐H1299 (*middle panel*), and NCI‐H460 (*right panel*) cells were transfected with *pGL3‐Noxa‐N1* plasmid or *pGL3‐Basic* vector. The *Noxa* promoter activity was measured by dual luciferase reporter assays. Firefly luciferase readings were normalized to *Renilla* luciferase to correct for transfection efficiency. The *Noxa* promoter‐driven luciferase activities were expressed as fold induction over the activity of *pGL3‐Basic* vector. Data represent mean ± SD from two independent experiments performed in triplicate. *P < 0.05, **P < 0.01, significant difference compared with the shGFP control cells. D and E, Overexpession of Bmi1 diminishes the *Noxa* promoter activity in NSCLC cells. Expression level of Bmi1 was examined by Western blot analysis with specific antibodies. β‐actin was used as a loading control (D). NCI‐H23 (E, *left panel*), NCI‐H1299 (E, *middle panel*), and NCI‐H460 (E, *right panel*) cells were co‐transfected *pGL3‐Noxa‐N1* plasmid or *pGL3‐Basic* vector along with *pT3‐EF1a‐Bmi1* or empty vector for 48 hours as described in Materials and Methods. The *Noxa* promoter activity was measured by dual luciferase reporter assays. Firefly luciferase readings were normalized to *Renilla* luciferase to correct for transfection efficiency. The *Noxa* promoter‐driven luciferase activities were expressed as fold induction over the activity of *pGL3‐Basic* vector. Data represent mean ± SD from two independent experiments performed in triplicate. *P < 0.05, significant difference compared with the empty vector‐transfected control cells

Since suppression of Bmi1 level by deguelin was accompanied with increased Noxa level and apoptosis in NSCLC cells (Figure 4A, *left*), we surmised that deguelin induced cell apoptosis through Bmi1‐regulated Noxa induction. Then, we used *pGL3‐Noxa‐N1* reporter, in which a *luciferase* reporter gene was linked to the *Noxa* promoter and the expression of luciferase was driven by the *Noxa* promoter, to assess the effect of deguelin on the *Noxa* promoter activity. Results showed that expression of luciferase, the indicative of *Noxa* promoter activity, was significantly increased upon deguelin treatment in NCI‐H23, NCI‐H1299 and NCI‐H460 cells (Figure 5A). Since pharmacological inhibition of Bmi1 by deguelin was accompanied with Noxa upregulation in NSCLC cells (Figure 4A, *left*), we speculated that suppression of Bmi1 by RNA interference might also increase Noxa level. Knockdown of Bmi1 (Figure 5B) led to increases in both Noxa protein level (Figure 5B) and luciferase expression driven by the *Noxa* promoter (Figure 5C), indicating that Bmi1 regulated the induction of *Noxa* gene in these NSCLC cells. To further determine whether Bmi1 directly activates the *Noxa* promoter, *pGL3‐Noxa‐N1* reporter was co‐transfected with the Bmi1 expression plasmid to assess the contribution of Bmi1 to the *Noxa* promoter activity. Results demonstrated that overexpression of Bmi1 (Figure 5D) significantly decreased the *Noxa* promoter activity in NCI‐H23, NCI‐H1299, and NCI‐H460 cells (Figure 5E). Collectively, these data suggest that deguelin partially induces apoptosis through downregulation of Bmi1 and induction of Noxa in NSCLC cells.

### 3.6 Deguelin inhibits the directly binding of Bmi1 to the Noxa gene locus

**Figure 6:**
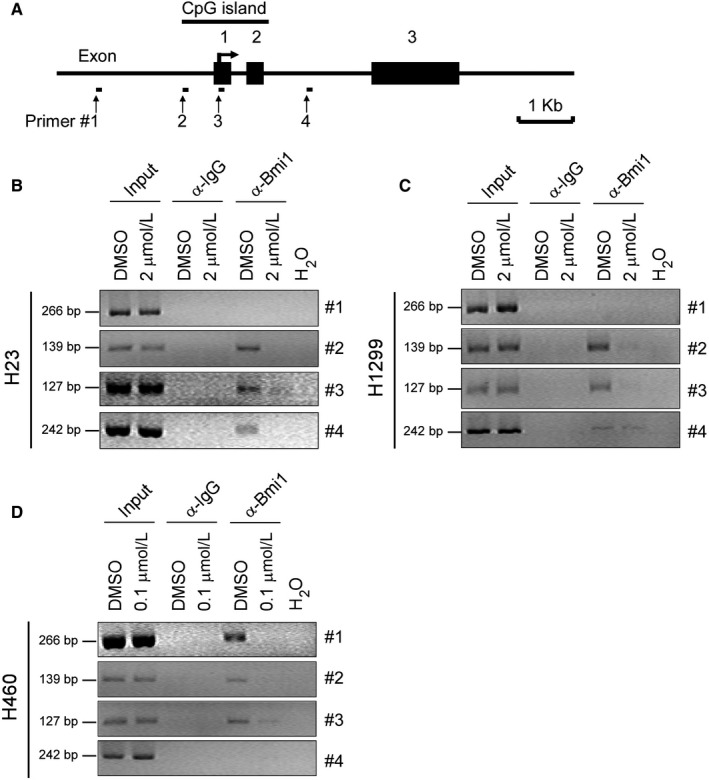
Deguelin inhibits the binding of Bmi1 to the *Noxa* gene locus in NSCLC cells. A, Schematic representation of the *Noxa* locus and the location of primers (#1 to #4) used in ChIP experiments and exons are indicated. B‐D, NCI‐H23 (B), NCI‐H1299 (C), and NCI‐H460 (D) cells were treated with DMSO or the indicated concentrations of deguelin for 48 hours and subjected to ChIP assays with an antibody against Bmi1 or normal rabbit IgG. The precipitated DNA fragments were subjected to PCR analysis to test for the presence of sequences corresponding to the *Noxa* gene locus. Input material (10%) was shown for comparison

To demonstrate the effect of deguelin on the interaction of Bmi1 with the *Noxa* promoter in vivo, we performed ChIP assays. NSCLC cells were treated with DMSO or deguelin for 48 hours, chromatin was immunoprecipitated and the binding of Bmi1 to *Noxa* specific genomic regions was analyzed by PCR using primers designed around the *Noxa* promoter region (Figure 6A, Figure S4, Table S1). ChIP assays with NCI‐H23, NCI‐H1299, and NCI‐H460 cells showed that Bmi1 interacted with the *Noxa* gene locus (Figure 6B‐D). Notably, accumulation of Bmi1 was always observed within the CpG islands (Figure 6A and Figure S4A) of *Noxa* gene in all three NSCLC cell lines (Figure 6B‐D, #2 and #3). Also, deguelin significantly disrupted the interaction of Bmi1 with the *Noxa* locus (Figure 6B‐D). These results suggest that deguelin directly targets Bmi1 to inhibit its binding to the *Noxa* promoter and relieves Bmi1‐mediated Noxa repression to increase Noxa expression, leading to deguelin‐induced apoptosis in NSCLC cells.

## 4 DISCUSSION

Although deguelin has been reported to induce apoptosis in various cancer cells, the molecular details of this regulation remain to be fully disclosed. In this report, we showed that BH3‐only protein Noxa is up‐regulated during deguelin‐induced apoptosis in a panel of NSCLC cell lines, which is independent of p53. Moreover, the upregulation of Noxa is consistently accompanied by deguelin‐reduced Bmi1, an important component of the PRC1. We further confirmed that deguelin attenuates the binding of Bmi1 to the *Noxa* promoter and removes Bmi1‐caused repression, thus resulting in Noxa induction. This study has revealed a novel mechanism by which deguelin activates the apoptotic machinery in NSCLC cells.

Noxa prefers to localize in mitochondria^24^ and displays quite variable pro‐apoptotic potential in various cell types. For example, overexpression of Noxa results in significant cell death in human Saos2 cells,^24^ but in many other cell types, including MEF cells, it proved poorly apoptotic function.^48^ Noxa binds preferentially to Mcl‐1 and weakly to A1^25^ and exerts its pro‐apoptotic function mainly by neutralizing these proteins, facilitating activation of Bax and/or Bak proteins. Noxa has been implicated to disrupt Mcl‐1/Bak complex, displace Bak from the Mcl‐1/Bak complex, cause Bak being free for oligomerization and induce apoptosis in diverse cell types, such as HEK‐293T, multiple myeloma, and B‐cell lymphomas.^25^ Moreover, Noxa‐induced cell death is more relevant for apoptosis induction in malignant or highly proliferating vs primary or differentiated cells, suggesting its importance as target for therapy.^25^ Our results showed that z‐VAD‐fmk treatment can't significantly up‐regulate Mcl‐1 protein level (Figure 4E), which suggested that Mcl‐1 suppression may also contribute to Noxa‐induced apoptosis in deguelin‐treated NSCLC cells, but not a consequence of deguelin‐induced apoptosis. Similarly, deguelin inhibited Mcl‐1 expression in B‐cell chroniclymphocytic leukemia cells^49^ and prostate cancer cells^42^ to promote apoptosis. Deguelin reportedly binds directly to Mcl‐1 in the hydrophobic grooves.^50^ In addition, Noxa can bind to Mcl‐1 and trigger its proteasomal degradation.^51^ Therefore, we speculated that the mechanisms of downregulation of Mcl‐1 in present models might result from: (a) directly inhibited by deguelin; (b) the rising level of Noxa causes degradation of Mcl‐1 for proteasomal degradation; (c) both deguelin itself and deguelin‐induced Noxa regulate Mcl‐1 expression level. The more detailed mechanism(s) merits further investigation.

Although Noxa was originally described as a p53‐regulated gene, it can also be modulated in a p53‐independent manner.^25^ To determine whether the *p53* status affected Noxa induction after deguelin treatment, *p53* mutant NCI‐H23, *p53*‐null NCI‐H1299, and *p53* wild‐type NCI‐H460 were selected for further experiments. Noxa protein increased by deguelin in *p53* mutant NCI‐H23 cells, *p53*‐null NCI‐H1299 cells or *p53* wild‐type NCI‐H460 cells was not significantly affected (Figure 4A, *left*). These results suggested that induction of Noxa by deguelin is irrespective of the *p53* status in these NSCLC cell lines.

Our results indicate that deguelin increases Noxa and decreases Mcl‐1 simultaneously, which means a consistent elevation of Noxa/Mcl‐1 ratio induces apoptosis (Figure 4A, *left*). The result suggested that the ratio of Noxa to Mcl‐1 may regulate cell apoptosis decision to undergo apoptosis vs survival upon deguelin treatment. These results are similar to the finding that pemetrexed induced Noxa upregulation and Mcl‐1 downregulation concurrently, which accelerates pemetrexed‐induced apoptosis in NSCLC cells.^52^ Interestingly, in the current study, we found that deguelin promoted Bax and Noxa mitochondrial protein levels. Overexpression of Bim1 suppressed deguelin‐induced Bax and Noxa mitochondrial localization (Figure 4F) and attenuated the release of cytochrome c from mitochondria. These results indicated that rectification of Bmi1/Noxa axis disrupts the mitochondrial potential and results in apoptosis in deguelin‐treated NSCLC cells.

Deguelin is reportedly an Hsp90 inhibitor. It directly binds to the ATP‐binding pocket of Hsp90, interferes with Hsp90 chaperone function, decreases the expression of many Hsp90 client proteins, and induces apoptosis in cancer cells, which reduces tumour growth.^53^ Although deguelin has been mentioned as an Akt inhibitor,^32^, ^34^ whether Akt is a direct target of deguelin and the mechanism of how deguelin inhibits Akt activity remains undisclosed. On the molecular level, it has also been reported that deguelin inhibits several other signalling pathways (such as IKK/IκBα/NF‐κB pathway^34^) and a number of proteins (such as COX2^36^). However, the direct target(s) of deguelin remains to be further clarified.

PcG genes control transcription through remodelling chromatin structures by constituting polycomb repressive complexes enabling DNA methylation at CpG islands.^54^ Yamashita et al^13^ observed that Bmi1 represses the *Noxa* gene expression and is required for DNA CpG islands methylation of the *Noxa* gene locus. Binding of Bmi1 to the *Noxa* locus depends on the methylation status of the promoter because knockdown of the methyltransferase Dnmt1 resulted in a decreased binding of Bmi1 to the *Noxa* promoter and an increased *Noxa* mRNA expression. Our results indicated that though the binding profiles of Bmi1 to the *Noxa* locus displayed difference to some extent in three NSCLC cell lines tested, CpG islands in the *Noxa* promoter are the preferable regions for Bmi1 binding (Figure 6). However, whether binding of Bmi1 to the *Noxa* promoter depends on the methylation status of the promoter and affects Noxa expression or not, needs to be further investigated.

Our results demonstrated that treatment with various concentrations of deguelin had no effect on trimethylation of H3K9 and H3K27 (Figure S3), which are linked to gene repression.^55^ Moreover, ChIP results indicated that deguelin did not inhibit the binding of H3K27me3 to the *Noxa* promoter (Figure S4B and C). H3K27 methylation was reportedly served as a binding site for the recruitment of PRC1 complex.^13^, ^56^ In addition, the level of H3K27 tri‐methylation was significantly decreased at the *Noxa* gene locus but was not affected at the *Ink4a* gene locus, another target gene of Bmi1, in the *Bmi1* ^−/−^ cells.^13^ Based on these results, deguelin did not inhibit the level of H3K27me3 as well as the binding of H3K27me3 to *Noxa* promoter. Thus, we speculated that deguelin did not dissociate Bmi1 from all sites on chromatin but directly targeted Bmi1 to inhibit its binding to the *Noxa* promoter, and relieved Bmil‐mediated Noxa repression, causing increased expression of Noxa. However, there is still a possibility that deguelin decreases the binding activity of Bmi1 on the promoter of other potential target genes under distinct conditions.

Our results indicated that H460 cells were far more sensitive to deguelin. Chemosensitivity is a multi‐factored phenotype, which could be influenced by various molecular determinants (i.e., DNA, RNA, or protein). In other words, gene polymorphisms, gene expression alterations, protein expression, and modification differences among individuals and within individual cancers can influence drug sensitivity.^57^ The genetic or epigenetic changes in genes that regulate apoptosis, DNA repair and senescence affects their intrinsic sensitivity to chemotherapy, which contributes to their intrinsic quality of chemosensitivity, when challenged with chemotherapeutic agents.^58^ At the present time, our data were insufficient to elucidate the underlying mechanisms why H460 cells are so sensitive to deguelin. However, the detailed mechanism underlying the differences in chemotherapeutic responses of cancer cells merits further investigation.

In summary, natural compound deguelin suppresses the growth of NSCLC cells regardless of their p53 status, and this anti‐tumour activity is partly dependent on the downregulation of Bmi1 and thus circumventing Bmi1‐mediated Noxa repression, resulting in Noxa induction and finally leading to NSCLC cells apoptosis. Upregulation of Noxa by different therapeutic strategies, including phytochemicals, may provide novel and valuable strategies for certain malignancies treatment, including NSCLC.

## CONFLICT OF INTEREST

The authors have declared no conflicts of interest.

## References

[1] Langer CJ , Besse B , Gualberto A , et al. The evolving role of histology in the management of advanced non‐small‐cell lung cancer. J Clin Oncol. 2010;28:5311‐5320. 21079145 10.1200/JCO.2010.28.8126

[2] Goldstraw P , Crowley J , Chansky K , et al. The IASLC Lung Cancer Staging Project: proposals for the revision of the TNM stage groupings in the forthcoming (seventh) edition of the TNM Classification of malignant tumours. J Thorac Oncol. 2007;2:706‐714. 17762336 10.1097/JTO.0b013e31812f3c1a

[3] Hernandez‐Munoz I , Taghavi P , Kuijl C , et al. Association of BMI1 with polycomb bodies is dynamic and requires PRC2/EZH2 and the maintenance DNA methyltransferase DNMT1. Mol Cell Biol. 2005;25:11047‐11058. 16314526 10.1128/MCB.25.24.11047-11058.2005 PMC1316945

[4] Valk‐Lingbeek ME , Bruggeman SW , van Lohuizen M . Stem cells and cancer; the polycomb connection. Cell. 2004;118:409‐418. 15315754 10.1016/j.cell.2004.08.005

[5] Cui H , Hu B , Li T , et al. Bmi‐1 is essential for the tumorigenicity of neuroblastoma cells. Am J Pathol. 2007;170:1370‐1378. 17392175 10.2353/ajpath.2007.060754 PMC1829469

[6] Dovey JS , Zacharek SJ , Kim CF , et al. Bmi1 is critical for lung tumorigenesis and bronchioalveolar stem cell expansion. Proc Natl Acad Sci U S A. 2008;105:11857‐11862. 18697930 10.1073/pnas.0803574105 PMC2575250

[7] Maynard MA , Ferretti R , Hilgendorf KI , et al. Bmi1 is required for tumorigenesis in a mouse model of intestinal cancer. Oncogene. 2014;33:3742‐3747. 23955081 10.1038/onc.2013.333 PMC3931743

[8] Park IK , Morrison SJ , Clarke MF . Bmi1, stem cells, and senescence regulation. J Clin Invest. 2004;113:175‐179. 14722607 10.1172/JCI20800 PMC311443

[9] Song LB , Li J , Liao WT , et al. The polycomb group protein Bmi‐1 represses the tumor suppressor PTEN and induces epithelial‐mesenchymal transition in human nasopharyngeal epithelial cells. J Clin Invest. 2009;119:3626‐3636. 19884659 10.1172/JCI39374 PMC2786794

[10] Jagani Z , Wiederschain D , Loo A , et al. The Polycomb group protein Bmi‐1 is essential for the growth of multiple myeloma cells. Cancer Res. 2010;70:5528‐5538. 20530672 10.1158/0008-5472.CAN-09-4229

[11] Teshima K , Nara M , Watanabe A , et al. Dysregulation of BMI1 and microRNA‐16 collaborate to enhance an anti‐apoptotic potential in the side population of refractory mantle cell lymphoma. Oncogene. 2014;33:2191‐2203. 23686310 10.1038/onc.2013.177

[12] Kimura M , Takenobu H , Akita N , et al. Bmi1 regulates cell fate via tumor suppressor WWOX repression in small‐cell lung cancer cells. Cancer Sci. 2011;102:983‐990. 21276135 10.1111/j.1349-7006.2011.01891.x

[13] Yamashita M , Kuwahara M , Suzuki A , et al. Bmi1 regulates memory CD4 T cell survival via repression of the Noxa gene. J Exp Med. 2008;205:1109‐1120. 18411339 10.1084/jem.20072000 PMC2373843

[14] Glinsky GV , Berezovska O , Glinskii AB . Microarray analysis identifies a death‐from‐cancer signature predicting therapy failure in patients with multiple types of cancer. J Clin Invest. 2005;115:1503‐1521. 15931389 10.1172/JCI23412 PMC1136989

[15] Kim JH , Yoon SY , Kim CN , et al. The Bmi‐1 oncoprotein is overexpressed in human colorectal cancer and correlates with the reduced p16INK4a/p14ARF proteins. Cancer Lett. 2004;203:217‐224. 14732230 10.1016/j.canlet.2003.07.009

[16] Mihic‐Probst D , Kuster A , Kilgus S , et al. Consistent expression of the stem cell renewal factor BMI‐1 in primary and metastatic melanoma. Int J Cancer. 2007;121:1764‐1770. 17597110 10.1002/ijc.22891

[17] Wang H , Pan K , Zhang HK , et al. Increased polycomb‐group oncogene Bmi‐1 expression correlates with poor prognosis in hepatocellular carcinoma. J Cancer Res Clin Oncol. 2008;134:535‐541. 17917742 10.1007/s00432-007-0316-8 PMC12161612

[18] Vonlanthen S , Heighway J , Altermatt HJ , et al. The bmi‐1 oncoprotein is differentially expressed in non‐small cell lung cancer and correlates with INK4A‐ARF locus expression. Br J Cancer. 2001;84:1372‐1376. 11355949 10.1054/bjoc.2001.1791 PMC2363629

[19] Ferretti R , Bhutkar A , McNamara MC , et al. BMI1 induces an invasive signature in melanoma that promotes metastasis and chemoresistance. Genes Dev. 2016;30:18‐33. 26679841 10.1101/gad.267757.115 PMC4701976

[20] Vrzalikova K , Skarda J , Ehrmann J , et al. Prognostic value of Bmi‐1 oncoprotein expression in NSCLC patients: a tissue microarray study. J Cancer Res Clin Oncol. 2008;134:1037‐1042. 18264721 10.1007/s00432-008-0361-y PMC12160758

[21] Li DW , Tang HM , Fan JW , et al. Expression level of Bmi‐1 oncoprotein is associated with progression and prognosis in colon cancer. J Cancer Res Clin Oncol. 2010;136:997‐1006. 20024662 10.1007/s00432-009-0745-7 PMC11827799

[22] Wu Z , Min L , Chen D , et al. Overexpression of BMI‐1 promotes cell growth and resistance to cisplatin treatment in osteosarcoma. PLoS ONE. 2011;6:e14648. 21311599 10.1371/journal.pone.0014648 PMC3032734

[23] Alajez NM , Shi W , Hui AB , et al. Targeted depletion of BMI1 sensitizes tumor cells to P53‐mediated apoptosis in response to radiation therapy. Cell Death Differ. 2009;16:1469‐1479. 19575017 10.1038/cdd.2009.85

[24] Oda E , Ohki R , Murasawa H , et al. Noxa, a BH3‐only member of the Bcl‐2 family and candidate mediator of p53‐induced apoptosis. Science. 2000;288:1053‐1058. 10807576 10.1126/science.288.5468.1053

[25] Ploner C , Kofler R , Villunger A . Noxa: at the tip of the balance between life and death. Oncogene. 2008;27(Suppl 1):S84‐S92. 19641509 10.1038/onc.2009.46 PMC3272398

[26] Schuler M , Green DR . Mechanisms of p53‐dependent apoptosis. Biochem Soc Trans. 2001;29:684‐688. 11709054 10.1042/0300-5127:0290684

[27] Nikiforov MA , Riblett M , Tang WH , et al. Tumor cell‐selective regulation of NOXA by c‐MYC in response to proteasome inhibition. Proc Natl Acad Sci U S A. 2007;104:19488‐19493. 18042711 10.1073/pnas.0708380104 PMC2148316

[28] Kim JY , Ahn HJ , Ryu JH , et al. BH3‐only protein Noxa is a mediator of hypoxic cell death induced by hypoxia‐inducible factor 1alpha. J Exp Med. 2004;199:113‐124. 14699081 10.1084/jem.20030613 PMC1887730

[29] Lallemand C , Blanchard B , Palmieri M , et al. Single‐stranded RNA viruses inactivate the transcriptional activity of p53 but induce NOXA‐dependent apoptosis via post‐translational modifications of IRF‐1, IRF‐3 and CREB. Oncogene. 2007;26:328‐338. 16832344 10.1038/sj.onc.1209795

[30] Hershko T , Ginsberg D . Up‐regulation of Bcl‐2 homology 3 (BH3)‐only proteins by E2F1 mediates apoptosis. J Biol Chem. 2004;279:8627‐8634. 14684737 10.1074/jbc.M312866200

[31] Boreddy SR , Srivastava SK . Deguelin suppresses pancreatic tumor growth and metastasis by inhibiting epithelial‐to‐mesenchymal transition in an orthotopic model. Oncogene. 2013;32:3980‐3991. 22986522 10.1038/onc.2012.413 PMC3530646

[32] Chun KH , Kosmeder JW 2nd , Sun S , et al. Effects of deguelin on the phosphatidylinositol 3‐kinase/Akt pathway and apoptosis in premalignant human bronchial epithelial cells. J Natl Cancer Inst. 2003;95:291‐302. 12591985 10.1093/jnci/95.4.291

[33] Li W , Gao F , Ma X , et al. Deguelin inhibits non‐small cell lung cancer via down‐regulating Hexokinases II‐mediated glycolysis. Oncotarget. 2017;8:32586‐32599. 28427230 10.18632/oncotarget.15937 PMC5464811

[34] Nair AS , Shishodia S , Ahn KS , et al. Deguelin, an Akt inhibitor, suppresses IkappaBalpha kinase activation leading to suppression of NF‐kappaB‐regulated gene expression, potentiation of apoptosis, and inhibition of cellular invasion. J Immunol. 2006;177:5612‐5622. 17015749 10.4049/jimmunol.177.8.5612

[35] Jin Q , Feng L , Behrens C , et al. Implication of AMP‐activated protein kinase and Akt‐regulated survivin in lung cancer chemopreventive activities of deguelin. Cancer Res. 2007;67:11630‐11639. 18089792 10.1158/0008-5472.CAN-07-2401

[36] Lee HY , Suh YA , Kosmeder JW , et al. Deguelin‐induced inhibition of cyclooxygenase‐2 expression in human bronchial epithelial cells. Clin Cancer Res. 2004;10:1074‐1079. 14871987 10.1158/1078-0432.ccr-0833-3

[37] Murillo G , Peng X , Torres KE , et al. Deguelin inhibits growth of breast cancer cells by modulating the expression of key members of the Wnt signaling pathway. Cancer Prev Res (Phila). 2009;2:942‐950. 19861542 10.1158/1940-6207.CAPR-08-0232

[38] Murillo G , Salti GI , Kosmeder JW 2nd , et al. Deguelin inhibits the growth of colon cancer cells through the induction of apoptosis and cell cycle arrest. Eur J Cancer. 2002;38:2446‐2454. 12460790 10.1016/s0959-8049(02)00192-2

[39] Yu X , Liang Q , Liu W , et al. Deguelin, an aurora B kinase inhibitor, exhibits potent anti‐tumor effect in human esophageal squamous cell carcinoma. EBioMedicine. 2017;26:100‐111. 29129699 10.1016/j.ebiom.2017.10.030 PMC5832566

[40] Oh SH , Woo JK , Jin Q , et al. Identification of novel antiangiogenic anticancer activities of deguelin targeting hypoxia‐inducible factor‐1 alpha. Int J Cancer. 2008;122:5‐14. 17764071 10.1002/ijc.23075

[41] Li M , Yu X , Li W , et al. Deguelin suppresses angiogenesis in human hepatocellular carcinoma by targeting HGF‐c‐Met pathway. Oncotarget. 2017;9:152‐166. 29416603 10.18632/oncotarget.22077 PMC5787453

[42] Thamilselvan V , Menon M , Thamilselvan S . Anticancer efficacy of deguelin in human prostate cancer cells targeting glycogen synthase kinase‐3 beta/beta‐catenin pathway. Int J Cancer. 2011;129:2916‐2927. 21472727 10.1002/ijc.25949

[43] Liu H , Duan Z , Zheng H , et al. EBV‐encoded LMP1 upregulates Igkappa 3′enhancer activity and Igkappa expression in nasopharyngeal cancer cells by activating the Ets‐1 through ERKs signaling. PLoS ONE. 2012;7:e32624. 22396784 10.1371/journal.pone.0032624 PMC3291551

[44] Liu H , Hwang J , Li W , et al. A derivative of chrysin suppresses two‐stage skin carcinogenesis by inhibiting mitogen‐ and stress‐activated kinase 1. Cancer Prev Res (Phila). 2014;7:74‐85. 24169959 10.1158/1940-6207.CAPR-13-0133 PMC3947278

[45] Sheng Y , Li W , Zhu F , et al. 3,6,2′,4′,5′‐Pentahydroxyflavone, an orally bioavailable multiple protein kinase inhibitor, overcomes gefitinib resistance in non‐small cell lung cancer. J Biol Chem. 2012;289:28192‐28201. 10.1074/jbc.M114.593475 PMC4192475 25122774

[46] Dell'Eva R , Ambrosini C , Minghelli S , et al. The Akt inhibitor deguelin, is an angiopreventive agent also acting on the NF‐kappaB pathway. Carcinogenesis. 2007;28:404‐413. 16952909 10.1093/carcin/bgl162

[47] Holubec H , Payne CM , Bernstein H , et al. Assessment of apoptosis by immunohistochemical markers compared to cellular morphology in ex vivo‐stressed colonic mucosa. J Histochem Cytochem. 2005;53:229‐235. 15684335 10.1369/jhc.4A6386.2005

[48] Chen L , Willis SN , Wei A , et al. Differential targeting of prosurvival Bcl‐2 proteins by their BH3‐only ligands allows complementary apoptotic function. Mol Cell. 2005;17:393‐403. 15694340 10.1016/j.molcel.2004.12.030

[49] Geeraerts B , Vanhoecke B , Vanden Berghe W , et al. Deguelin inhibits expression of IkappaBalpha protein and induces apoptosis of B‐CLL cells in vitro. Leukemia. 2007;21:1610‐1618. 17568818 10.1038/sj.leu.2404788

[50] Hafeez S , Urooj M , Saleem S , et al. BAD, a proapoptotic protein, escapes ERK/RSK phosphorylation in deguelin and siRNA‐treated HeLa cells. PLoS ONE. 2016;11:e0145780. 26745145 10.1371/journal.pone.0145780 PMC4706341

[51] Willis SN , Chen L , Dewson G , et al. Proapoptotic Bak is sequestered by Mcl‐1 and Bcl‐xL, but not Bcl‐2, until displaced by BH3‐only proteins. Genes Dev. 2005;19:1294‐1305. 15901672 10.1101/gad.1304105 PMC1142553

[52] Yan J , Zhong N , Liu G , et al. Usp9x‐ and Noxa‐mediated Mcl‐1 downregulation contributes to pemetrexed‐induced apoptosis in human non‐small‐cell lung cancer cells. Cell Death Dis. 2014;5:e1316. 24991768 10.1038/cddis.2014.281 PMC4123075

[53] Oh SH , Woo JK , Yazici YD , et al. Structural basis for depletion of heat shock protein 90 client proteins by deguelin. J Natl Cancer Inst. 2007;99:949‐961. 17565155 10.1093/jnci/djm007

[54] Wang H , Wang L , Erdjument‐Bromage H , et al. Role of histone H2A ubiquitination in Polycomb silencing. Nature. 2004;431:873‐878. 15386022 10.1038/nature02985

[55] Ellis L , Atadja PW , Johnstone RW . Epigenetics in cancer: targeting chromatin modifications. Mol Cancer Ther. 2009;8:1409‐1420. 19509247 10.1158/1535-7163.MCT-08-0860

[56] Gibbons RJ . Histone modifying and chromatin remodelling enzymes in cancer and dysplastic syndromes. Hum Mol Genet. 2005;14:R85‐R92. 15809277 10.1093/hmg/ddi106

[57] Auman JT , McLeod HL . Cancer pharmacogenomics: DNA genotyping and gene expression profiling to identify molecular determinants of chemosensitivity. Drug Metab Rev. 2008;40:303‐315. 18464047 10.1080/03602530801952427

[58] Savage P , Stebbing J , Bower M , Crook T . Why does cytotoxic chemotherapy cure only some cancers? Nat Clin Pract Oncol. 2009;6:43‐52. 18982000 10.1038/ncponc1260

