# Supplementary figures and images for: Repression of Noxa by Bmi1 contributes to deguelin‐induced apoptosis in non‐small cell lung cancer cells
Source: J Cell Mol Med. 2018 Sep 25;22(12):6213–27. doi: 10.1111/jcmm.13908 (PMC6237602; doi:10.1111/jcmm.13908)

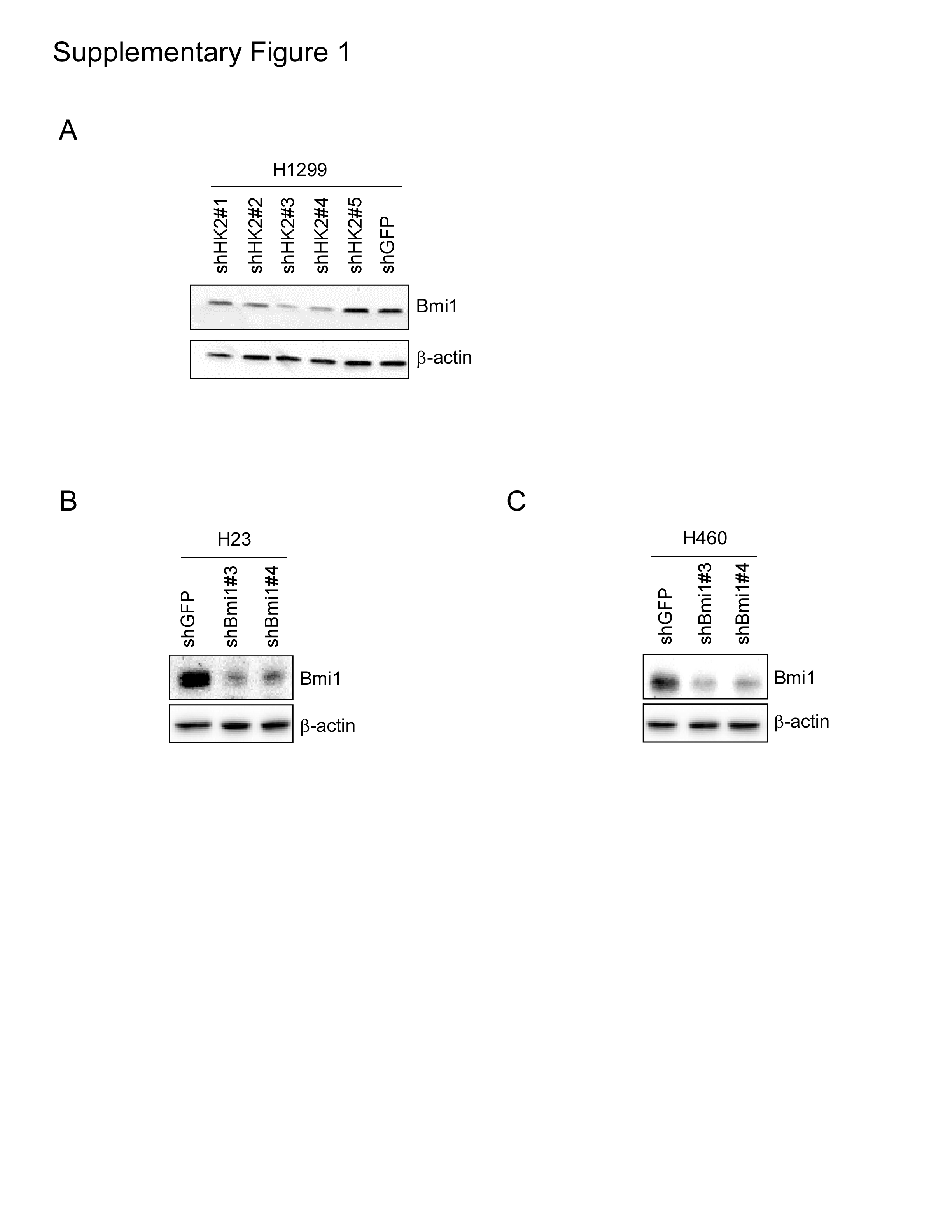

Supplement: Supplementary file 1 [file JCMM-22-6213-s001.jpg]

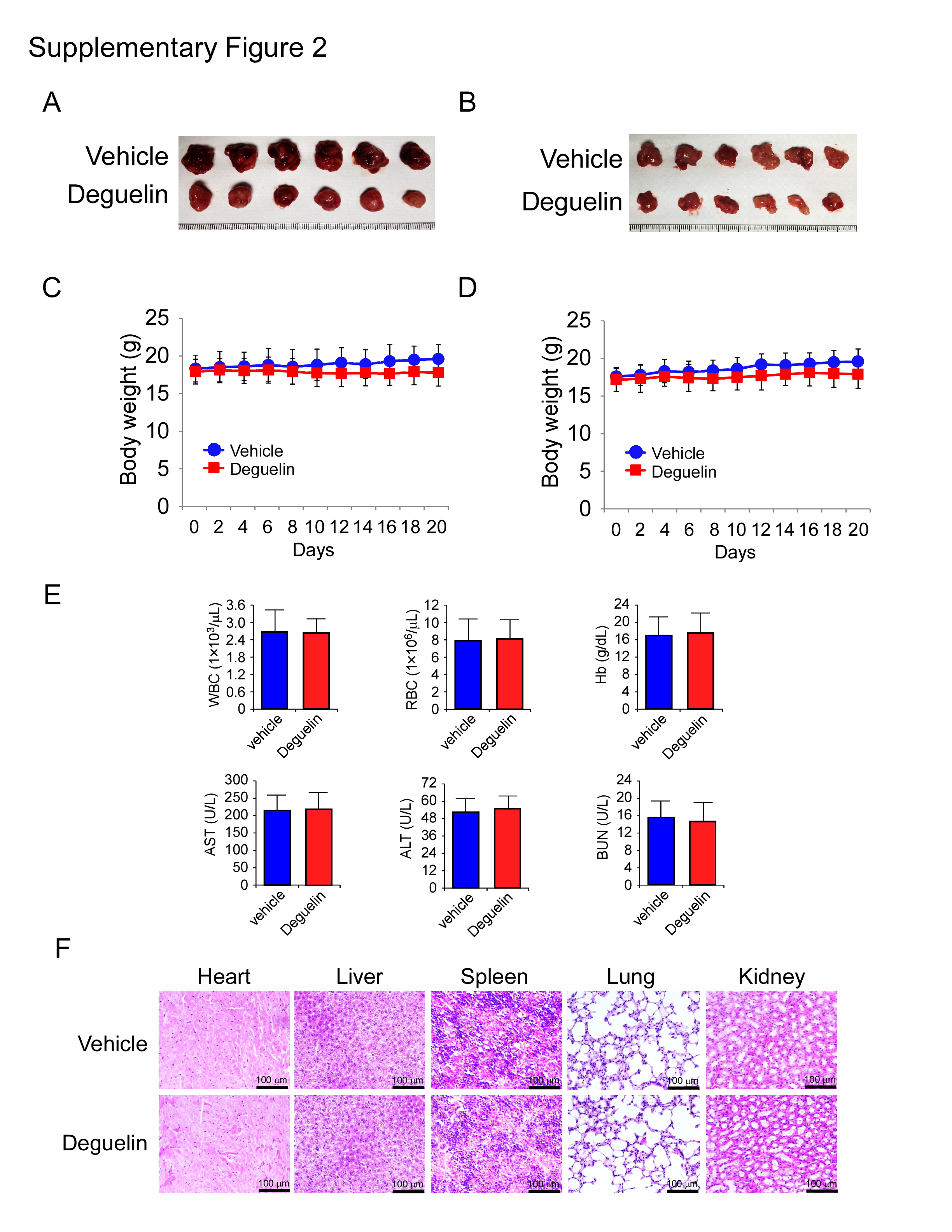

Supplement: Supplementary file 2 [file JCMM-22-6213-s002.jpg]

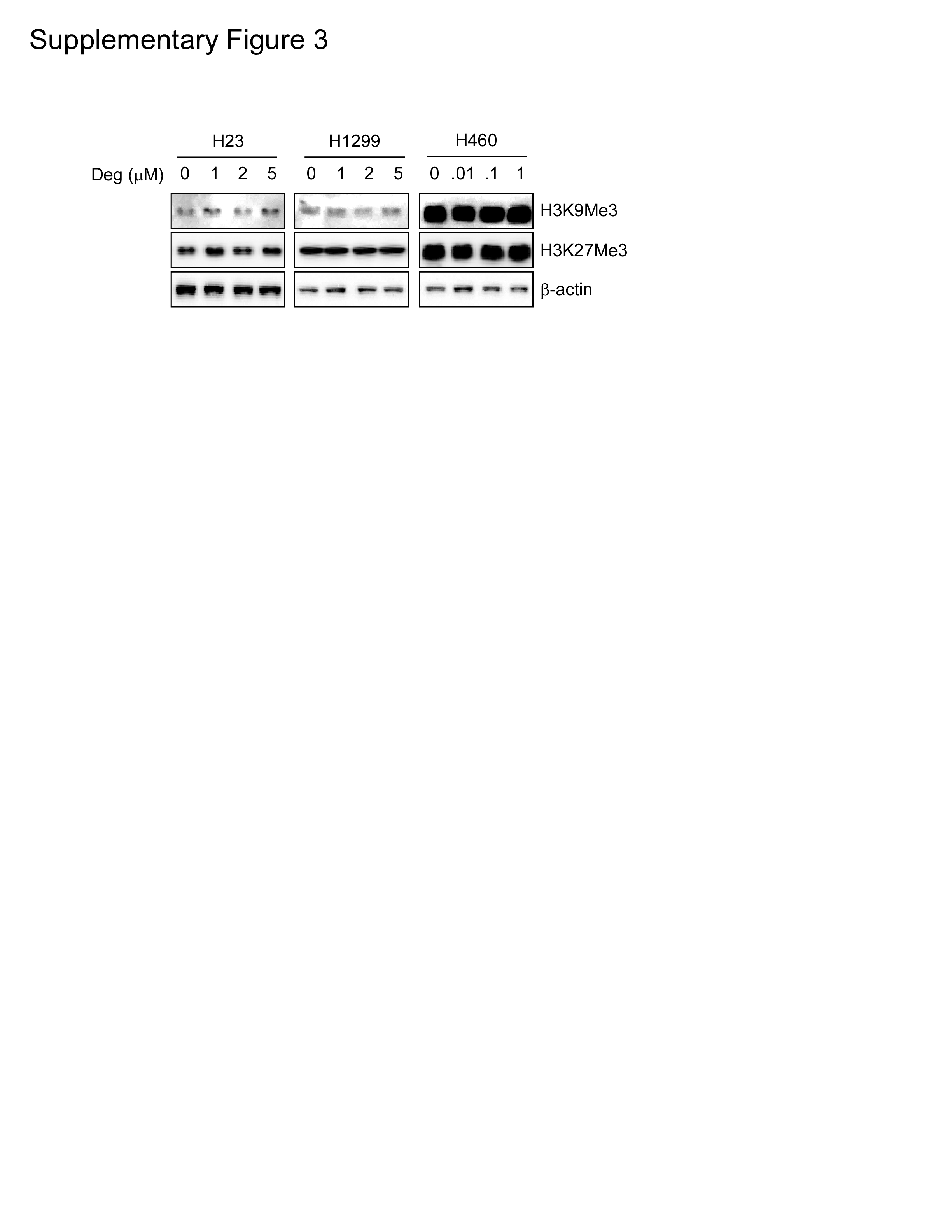

Supplement: Supplementary file 3 [file JCMM-22-6213-s003.jpg]

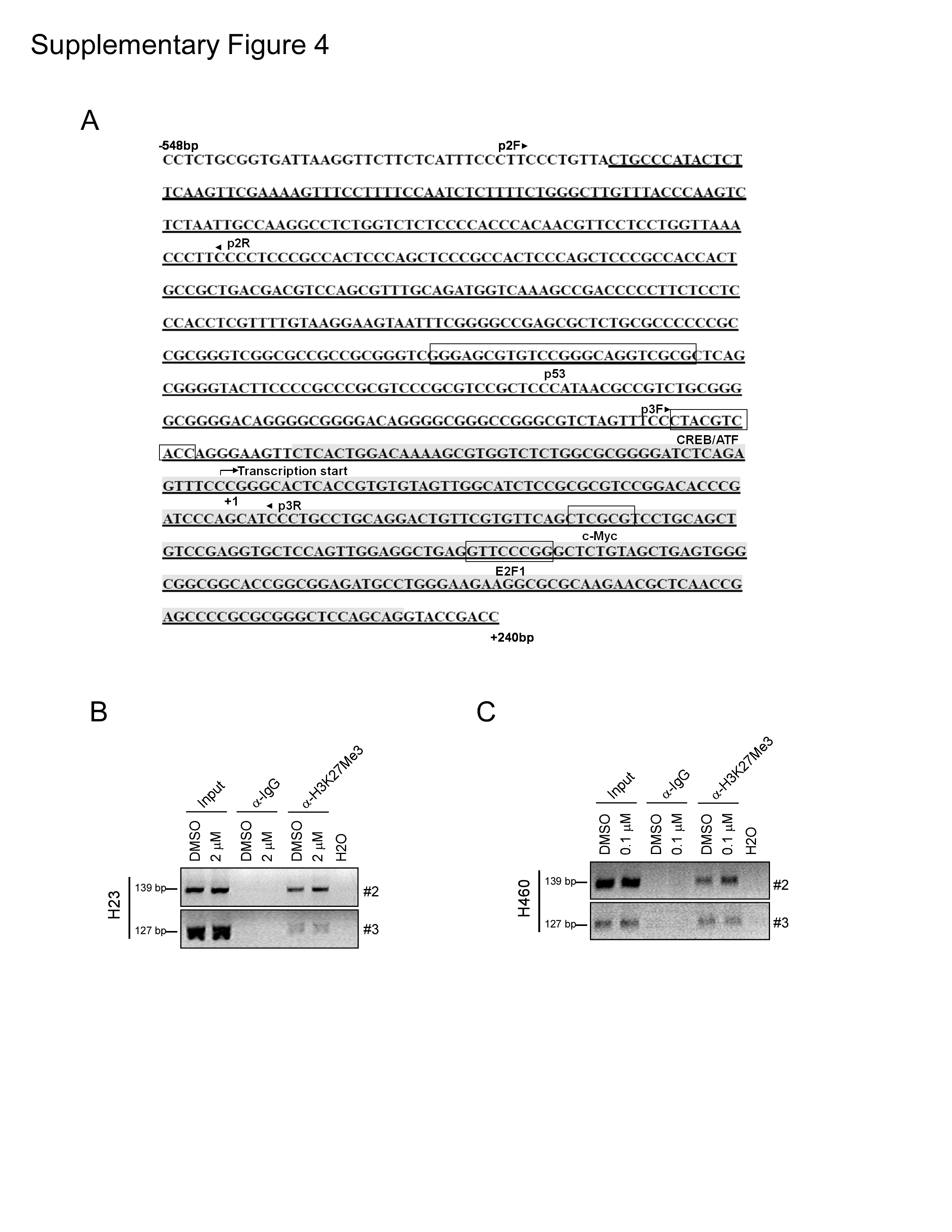

Supplement: Supplementary file 4 [file JCMM-22-6213-s004.jpg]
