# Supplementary material for: Repression of Noxa by Bmi1 contributes to deguelin‐induced apoptosis in non‐small cell lung cancer cells
Source: J Cell Mol Med. 2018 Sep 25;22(12):6213–27. doi: 10.1111/jcmm.13908 (PMC6237602; doi:10.1111/jcmm.13908)
Supplement: Supplementary file 5 [file JCMM-22-6213-s005.doc]

**Table S1 Primers for amplification of *Noxa* promoter by PCR**

| **Primer** | **Sense sequence (5'to3')** | **Antisense sequence (5'to3')** | **Size (bp)** |
| --- | --- | --- | --- |
| #1 | GCTGAATCTGACCCATGTCC | AAATACGCACCCTGAAATGC | 266 |
| #2 | TCCCTGTTACTGCCCATACTCT | GAAGGGTTTAACCAGGAGGAAC | 139 |
| #3 | CCTACGTCACCAGGGAAGTT | GATGCTGGGATCGGGTGT | 127 |
| #4 | CGAAAGACCTCAAGCTGCTC | CCAATCCATTGCCTTTATGG | 242 |
